# Synthesis, Anticancer Potential and Comprehensive Toxicity Studies of Novel Brominated Derivatives of Bacterial Biopigment Prodigiosin from *Serratia marcescens* ATCC 27117

**DOI:** 10.3390/molecules27123729

**Published:** 2022-06-09

**Authors:** Jelena Lazic, Sanja Skaro Bogojevic, Sandra Vojnovic, Ivana Aleksic, Dusan Milivojevic, Martin Kretzschmar, Tanja Gulder, Milos Petkovic, Jasmina Nikodinovic-Runic

**Affiliations:** 1 Graduate School, University of Nova Gorica, SI-5000 Nova Gorica, Slovenia; jelena.lazic@student.ung.si; 2 Institute of Molecular Genetics and Genetic Engineering, University of Belgrade, 11000 Belgrade, Serbia; sanja.bogojevic@imgge.bg.ac.rs (S.S.B.); sandravojnovic@imgge.bg.ac.rs (S.V.); ivana_aleksic@imgge.bg.ac.rs (I.A.); dusan.milivojevic@imgge.bg.ac.rs (D.M.); 3 Institute of Organic Chemistry, Leipzig University, 04103 Leipzig, Germany; martin.kretzschmar@uni-leipzig.de (M.K.); tanja.gulder@uni-leipzig.de (T.G.); 4 Faculty of Pharmacy, University of Belgrade, 11000 Belgrade, Serbia; milosp@pharmacy.bg.ac.rs

**Keywords:** prodigiosin, *Serratia*, halogenation, bromination, anticancer activity, embryotoxicity, zebrafish

## Abstract

Prodigiosins (prodiginines) are a class of bacterial secondary metabolites with remarkable biological activities and color. In this study, optimized production, purification, and characterization of prodigiosin (PG) from easily accessible *Serratia marcescens* ATCC 27117 strain has been achieved to levels of 14 mg/L of culture within 24 h. Furthermore, environmentally friendly bromination of produced PG was used to afford both novel mono- and dibrominated derivatives of PG. PG and its Br derivatives showed anticancer potential with IC_50_ values range 0.62–17.00 µg/mL for all tested cancer cell lines and induction of apoptosis but low selectivity against healthy cell lines. All compounds did not affect *Caenorhabditis* *elegans* at concentrations up to 50 µg/mL. However, an improved toxicity profile of Br derivatives in comparison to parent PG was observed in vivo using zebrafish (*Danio rerio*) model system, when 10 µg/mL applied at 6 h post fertilization caused death rate of 100%, 30% and 0% by PG, PG-Br, and PG-Br_2,_ respectively, which is a significant finding for further structural optimizations of bacterial prodigiosins. The drug-likeness of PG and its Br derivatives was examined, and the novel Br derivatives obey the Lipinski’s “rule of five”, with an exemption of being more lipophilic than PG, which still makes them good targets for further structural optimization.

## 1. Introduction

**Figure 1:**
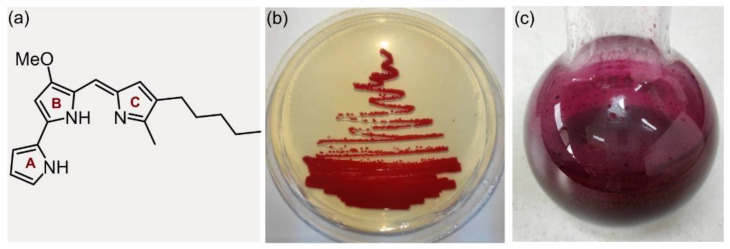
(**a**) Prodigiosin structure (A, B, and C rings); (**b**) red color of the prodigiosin-producing strain *S. marcescens* ATCC 27117 on a nutrient agar plate; (**c**) purified prodigiosin from the *S. marcescens* ATCC 27117 liquid culture.

Prodigiosins (prodiginines) are biologically active pyrrolylpyrromethene alkaloids, with the first records of their producing strains dating back to the VI century BC [1]. Prodigiosins derive their name from the miraculous (prodigious) events associated with their occurrence, and the producing strain was identified in 1819 for the first time, by a Venetian pharmacist Bartolomeo Bizio, attributing the organism to an episode of blood-red discoloration of polenta [2]. The first discovered pigment was prodigiosin (PG, Figure 1a) in 1902, and it was investigated for over 50 years until its structure was finally confirmed [3]. PG consists of three pyrrole rings (A, B and C, Figure 1a), where A and B rings are bridged in a bipyrrole unit and B and C rings are attached in a dipyrrin moiety, making the monopyrrole moiety (C ring) connected to the methoxy bipyrrole moiety (A and B rings) by a methylene bridge. Prodigiosins are formed in the later stages of bacterial growth, and the intense color of these secondary metabolite pigments can vary from red to purple, due to the presence of a conjugated system of double bonds (Figure 1b,c). Prodigiosins can be produced by both Gram-positive bacteria, e.g., *Streptomyces* spp., and Gram-negative bacteria, e.g., *Serratia* spp. [4].

*Serratia* spp. are ubiquitous, motile, rod-shaped, Gram-negative, facultative anaerobic bacteria belonging to the Enterobacteriaceae family [5]. They are used in agriculture for biocontrol purposes, since they secrete extracellular enzymes (chitinases, nucleases, lipases, proteases), surfactant serrawettin, and a variety of bioactive molecules (prodigiosins, carbapenems, antifungals…) [6]. Out of the 18 classified *Serratia* species so far, 4 have been described and characterized by their ability to produce PG: *S. marcescens* (Figure 1b), *S. nematodiphila*, *S. plymuthica*, and *S. rubidaea*. PG was first isolated from *S. marcescens* in 1954 [7]; however, producing strains are rarely deposited in the accessible culture collections. Although many studies have been performed using PG from *Serratia* strains isolated from natural sources [8,9], from patients [5], or upon genetic mutations [10], the optimization of the PG production and biological activity has rarely been reported for commercially available PG producers [11]. The method of choice for the estimation of the yield of PG is spectrophotometric measurement using the extinction coefficient ε_535_ of 0.159 L/(mg × cm) and a conversion to the concentration using the Lambert–Beer law [12]. However, due to different extinction coefficients used for these calculations and the purity of compounds, the PG concentration can be overestimated even by 270% [13]. Nevertheless, developing methods for direct isolation, purification, and measurement of the biopigment mass is still a challenging task.

**Figure 2:**
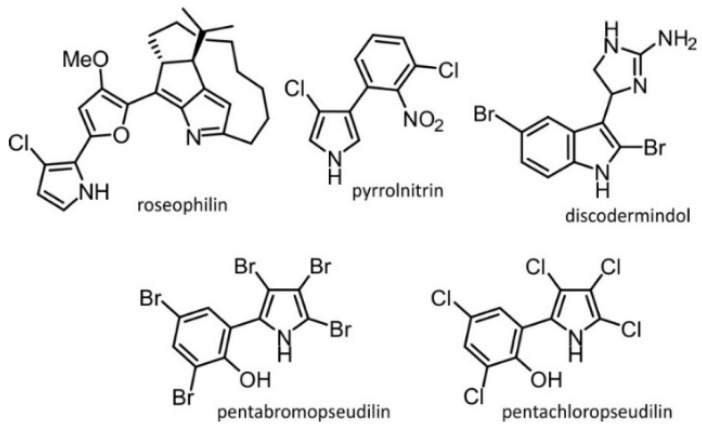
Structures of halogenated natural products produced by bacteria.

With the extensive research done regarding biological activities of PG, its anticancer [14,15], antiparasitic [16], antiprotozoal/antimalarial [17], immunosuppressive [18], antioxidative [9], antibacterial [19,20], and UV protective [21] properties were established. A halogenated natural product roseophilin (Figure 2), a relative of PG, with a methoxyfuran ring in the place of methoxypyrrol shows anticancer properties [22].

This is the case with several other notable examples of halogenated heterocycles isolated from bacteria, such as pyrrolnitrin [23] and discodermindol [24], while antibacterial properties have been noticed for pentabromopseudiline and pentachloropseudilin [23] (Figure 2).

Selective halogenation of natural products is becoming a popular research area due to the important pharmacological properties of organohalide molecules [25]. Since PG is a structurally complex heteroaromatic compound, regioselective and chemoselective halogenations are, in general, not easily accomplished. In addition, classical halogenations often require energy-demanding conditions, which often imply using toxic molecular halogens, such as chlorine gas or elemental bromine [26,27,28]. Classic chemical synthesis reactions, which would introduce carbon-halogen (C–X) bonds into the PG molecule are not mentioned in the literature. The incorporation of halogen entities in pharmacology leads has been predominantly performed to introduce higher target affinity, to increase lipophilicity and bioavailability, as well as to alter metabolic stability/pharmacokinetics and to lessen adverse effects [29]. In the search for new potential drug candidates, establishing drug-likeness is an important step as it allows for an assessment of the pharmacokinetic profiles of molecules based on the prediction of their solubility and permeability [30]. Due to the continued need for generating halogenated organic compounds, research focus is also on the development of more environmentally friendly and sustainable synthetic methods for generating C–X bonds [31,32]. These methodologies have been developed to be milder, environmentally acceptable, and allow for some control of the reaction outcome [31,33].

Although the anticancer potential of PG and other prodigiosins has been demonstrated through various modes of action, such as apoptosis [34], autophagy [15], cell cycle arrest [14], mitochondrial uncoupling [35], and migration inhibition [36], toxicity studies of PG are not readily available in the literature. The nematode *Caenorhabditis elegans* is a model system with well-known developmental stages [37] that can be utilized in anticancer drug discovery for toxicity, immunity, and pharmacogenetics studies, among others [38], so it represents a fitting model system for PG toxicity and safety research. In addition, zebrafish (*Danio rerio*) are commonly used in drug screens, as the transparency of embryos for up to 5 days post fertilization facilitates visualization of potential toxicities [39,40].

In this study, PG was produced in good yields from the commercially available *S. marcescens* ATCC 27117 strain, and for the first time, two halogenated derivatives were synthesized using a late-stage functionalization strategy and subsequently structurally characterized. Furthermore, brominated derivatives (Br derivatives) of PG were assessed in comparison to PG in terms of anticancer activity, mode of action, and cytotoxicity using both in vitro and in vivo model systems (*C. elegans* and *D. rerio*) and have proved to have more favorable toxicity profiles than PG. Drug-likeness of the novel Br derivatives was assessed by analyzing the Lipinski “rule of five” parameters, and they were found to violate only one of these rules namely being more lipophilic than the parent PG (logP > 5).

## 2. Results and Discussion

Since the discovery of PG, numerous synthetic routes and approaches have been established for PG and its analogs. In the first total synthesis by Rapoport and Willson, PG was successfully obtained by a 7-step protocol, but with an overall yield of 0.1% [41]. Many pathways for total PG synthesis are known today and have been refined over time with the development of the know-how of heterocyclic chemistry. Other total synthesis sequences for various prodigiosins family members (undecylprodigiosin, cycloprodigiosin, metacycloprodigiosin, streptorubin B, roseophilin…) are presented elsewhere [22,42], and the common ground in the total syntheses approaches is that they are costly and time-consuming (some involving 25 reaction steps), and the overall yields are usually low, which is why biotechnology and mutasynthesis have recently taken precedent [43,44,45].

### 2.1. S. marcescens ATCC 27117 Cultivation for Prodigiosin Production

**Figure 3:**
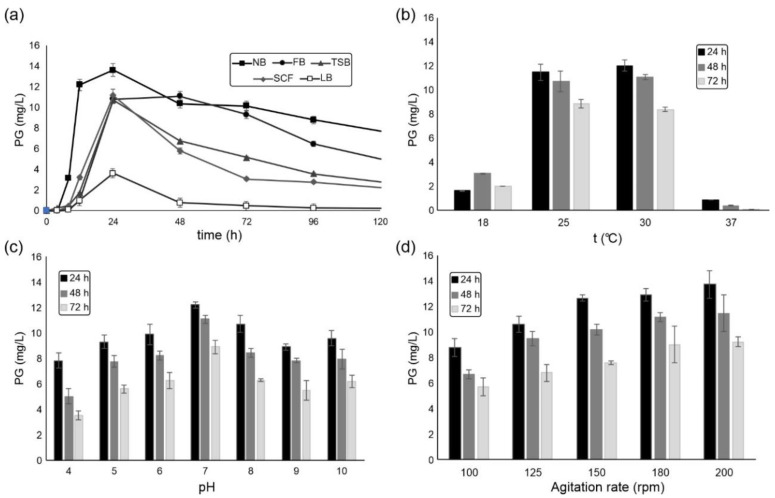
The influence of: (**a**) nutritional factors from different media; (**b**) temperature; (**c**) pH; (**d**) agitation rate, on the PG production in *S. marcescens* ATCC 27117 in flasks.

The initial experiments were designed to optimize the PG production in *S. marcescens* ATCC 27117 strain. Five media, commonly reported for PG production were assessed (Figure 3a). Cultivation of this strain in NB medium supported the highest PG concentration after 24 h (13.6 mg/L), followed by SCF (11.2 mg/L), FB (10.8 mg/L), TSB (10.7 mg/L), and finally LB medium (3.6 mg/L). Interestingly, extending the cultivation time to 48 h resulted in a slightly higher biopigment production only in FB medium (11.1 mg/L), which has the highest peptone content, while in other media a decrease in the biopigment concentration was noticed. Previously, peptone and yeast extract were found to be the best carbon and nitrogen sources for *S. marcescens* to produce PG [46], while other studies showed that glycerol plays an important role as a carbon source [47]. Beyond 48 h, prolonged cultivation time resulted in an overall PG decrease in each medium, due to the depletion of nutrients and possible degradation of PG. Indeed, it has been suggested that prodigiosin increases ATP production during culture lag phase and approximately doubles the stationary-phase cell yield; however, PG production is negatively associated with cellular ATP levels during high-rate, low-cell-density growth, indicating it is energy-spilling function during high rate, low cell density growth [48]. PG production in *S. marcescens* spp. varies from 5 mg/L [7] to the remarkable 49.5 g/L [49] by wild-type locally isolated strains.

The effects of physicochemical factors on the PG yield were also examined using the NB medium (Figure 3b–d). The favorable temperature for the cultivation was 30 °C at 180 rpm (Figure 3b), which is in a correlation with several previous studies, where the range of temperatures between 25–30 °C was found to be optimal, while temperatures higher than 30 °C have been known to interrupt the PG biosynthesis, most likely due to the biosynthetic enzyme inactivation [50]. The influence of pH was assessed at 30 °C at 180 rpm, and at pH 7 the highest production rate of PG was noticed after 24 h (Figure 3c), which agrees with the previously established results, slightly acidic to neutral pH conditions (5.5–7.0) have been appropriate for PG production in *Serratia* spp. [51]. The highest tested agitation rate of 200 rpm provided good aeration and afforded the highest PG concentration after 24 h (Figure 3d) and was selected for the larger-scale production.

In order to obtain enough of biopigment for derivatization reactions, cultivation of *S. marcescens* ATCC 27117 was carried out in the bioreactor, under the optimized cultivation conditions at pH 7, 28 °C, and 200 rpm. Fermentation was done in NB medium for 24 h, with the maximal PG production of 7.8 mg/L observed at 20 h (Figure S1). This yield was almost 2-fold lower than that achieved in flasks (Figure 3) but leaves room for further optimization. Indeed, usually fed-batch fermentation was found to be optimal for PG as well as some other biopigments production [10,46,52]. For example, by optimizing culture medium using brown sugar or cassava wastewater as low-cost carbon sources in a 5 L agitator bioreactor, yields of 8.1 g/L and 6.1 g/L were achieved, respectively [49,53].

Upon fermentation, the culture was centrifuged, the cells were separated from the supernatant, dried to a constant weight, and extracted with MeOH/HCl. Crude extract mass from 3.0 L fermentation in NB was 0.66 g/L, affording 7.2 mg/L (1.1%) of PG as a dark red solid, suggesting high efficiency of the extraction and purification procedure. The molecular formula of the purified pigment was determined by HR-LC-ESI-MS at *m/z* 324.2098 [M+H]^+^ to be C_20_H_25_N_3_O, corresponding to PG, and the chemical structure of purified PG was confirmed by ^1^H- and ^13^C-NMR spectroscopy (Figure S2), which was found to be identical to literature values [54].

### 2.2. Oxidative Prodigiosin Bromination

**Figure 4:**
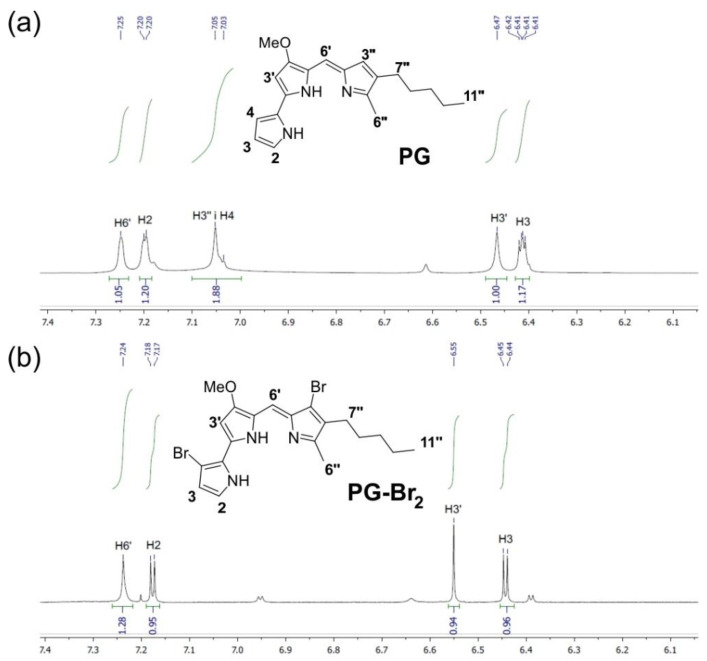
^1^H-NMR spectra of the aromatic region (7.40–6.00 ppm) of: (**a**) PG; (**b**) PG-Br_2_.

PG is a complex heteroaromatic natural product with several reactive positions (Figure 4a, positions 2, 3, 4, 3′, 6′, 3″), and reactions that would introduce carbon-halogen (C–X) bonds into the PG molecule are still not available in the literature. Due to the sensitivity of PG to light [55], its sensitivity to some solvents, and its instability at high temperatures [8], mild, late-stage functionalization is preferable. Inorganic acids, such as HBr, represent atom-economical sources of halide atoms, but they require an oxidant such as hydrogen peroxide (H_2_O_2_) to generate in situ a source of electrophilic “X^+^”, thus enabling electrophilic aromatic substitutions [32]. When hydrobromic acid is oxidized, elementary bromine is formed, which is disproportionated immediately in water, generating HOBr, the actual Br^+^ reagent. The slow generation of Br_2_ in situ is crucial [33]. These oxidative halogenation conditions exhibit high functional group tolerance and can be applied on a variety of electron-rich compounds, including PG.

**Scheme 1:**
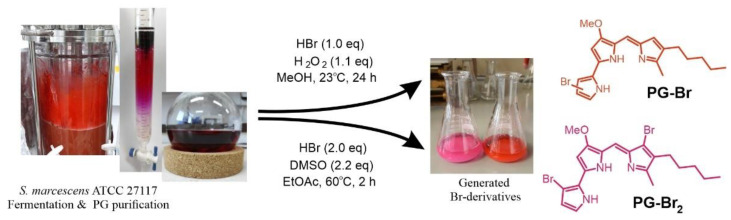
PG production, purification, and derivatization, affording two novel brominated derivatives.

After the process of fermentative production and chromatographic purification of PG, derivatization to obtain novel brominated derivatives was performed using hydrogen peroxide (H_2_O_2_) and dimethyl sulfoxide (DMSO) as green oxidants (Scheme 1). Bromination reaction of PG using H_2_O_2_ [31] as the oxidizing agent yielded a mixture of monobrominated products PG-Br, while DMSO as the oxidant [56] proceeded smoothly to give the dibrominated compound PG-Br_2_. Reaction yields were 81% of the monobrominated PG isomers and 63% of pure dibrominated PG.

Analysis of the ^1^H-NMR spectrum of the isolated PG-Br suggested that a mixture of two monobrominated products was obtained (Figures S3 and S4). The position of –Br in each isomer could not be determined, but it is evident that both isomers were acquired in approximately equal ratios. A careful comparison of the aromatic regions of the ^1^H-NMR spectra of PG and the compound PG-Br_2_ showed that the H-3″ and H-4 signals are absent in the spectrum of the dibrominated derivative (Figure 4b and Figure S5). Additional evidence for the positions of bromine atoms was the change in the H-3 and H-2 multiplicities. In the spectrum of PG in Figure 4a, H-3 signal appears in the form of a doublet of doublets, due to coupling with the H-2 and H-4 protons, while in the spectrum of the PG-Br_2_ in Figure 4b, this signal is in the form of a doublet due to coupling with the H-2 proton only. Similarly, H-2 signal in the PG spectrum appears as a multiplet, whereas in the dibrominated derivative it is in the form of a doublet, due to coupling with the vicinal H-3. Based on the positions of Br atoms in PG-Br_2_, it can be assumed that monobrominated isomers have the halogen either at position 4 or at the position 3″.

### 2.3. Anticancer Potential of Prodigiosin and Its Br Derivatives

PG has predominantly been explored as an anticancer compound, with promising potential in treating cancers of various cell types invading various organs, such as brain, lungs, liver, breasts, skin, colon, lungs, and others [57]. Prodigiosin has been screened in the 60-cell-line panel of the National Cancer Institute (NCI) and assessed for cytotoxic properties, with the average LC_50_ of 0.68 µg/mL [58]. PG was found to inhibit proliferation, migration, and invasion of a variety of cancer cell lines, including nasopharyngeal [36], breast [59], and lung [60]. Therefore, PG and two novel Br derivatives (PG-Br and PG-Br_2_) were used in cell viability assays on a healthy MRC-5 cell line, as well as a panel of A549, A375, MDA-MB-231, and HCT116 cancer cell lines (Table 1).

Pure PG exhibited excellent activity against all four carcinoma cell lines, with IC_50_ values between 0.62 and 1.30 µg/mL. Previously, the anticancer activity range of PG against A549 was found from as low as 0.03 µg/mL [61] to 3.23 µg/mL [15], so our finding of 1.30 µg/mL is in agreement with the literature. The melanoma cell line A375 was sensitive to PG at 0.70 µg/mL [62], which was similar to our finding of 1.25 µg/mL. Breast cancer MDA-MB-231 was found to be extremely sensitive to PG in the range from 0.02 µg/mL [59] up to 0.68 µg/mL [34]. The IC_50_ value reported previously for HCT116 colon cancer was 0.04 µg/mL after 72 h treatment [14], which is 10-fold lower in comparison to our findings (Table 1). However, PG was also highly toxic against healthy MRC-5 cells, and this could be one of the reasons that the natural PG has not reached the clinical practice, suggesting the need for derivatives with improved selectivity.

Both PG-Br and PG-Br_2_ showed lower cytotoxicity in comparison to PG, between 4- and 10-fold and 8- and 27-fold, respectively, including the activity against MRC-5 cells (Table 1). However, introduction of bromine(s) did not improve selectivity between healthy and cancer cell lines. While PG showed selectivity of 1.9 and 1.7 in the case of breast and colon cancer cells, the selectivity of 1.1 was present against HCT116 for PG-Br only. Overall, IC_50_ values for both Br derivatives were still considerable, and the novel compounds retained anticancer potential.

Bromination is expected to affect hydrophobicity of molecules, as well as facilitate transport across membranes and binding to hydrophobic pockets, especially since bromine substituents proved more hydrophobic than methoxy-moieties in vitro and in silico [63]; thus, shorter and longer exposure times were used to examine cytotoxicity in MRC-5 and HCT116 cells (Table 2 and Figure S6).

Prolonged exposure of MRC-5 cells to PG resulted in lower IC_50_ values over time (2.5-fold lower IC_50_ values of 72 h in comparison to 24 h exposure), while for the Br derivatives, the difference of 1.5-fold was observed between 24 h and 48 h, but longer treatment of up to 72 h had no further effect. In contrast, when HCT116 cells were exposed to PG and Br derivatives, the IC_50_ values of Br derivatives were much higher, and 11-fold lower IC_50_ values upon 72 h in comparison to 24 h exposure was observed for PG-Br, suggesting specific dynamics of its uptake and activity.

When healthy HFF (human foreskin fibroblasts) cells were treated with 0.36 µg/mL PG, growth inhibition was 76.16% and the IC_50_ was 0.15 µg/mL after 72 h of treatment [14]. When the effect of cycloprodigiosin (cPG) on breast cancer cell lines was studied, it turned out that healthy cells WI-38-40 (fibroblasts) and HBL-100 (breast epithelial cells) required higher doses of cPG for growth inhibition after 72 h exposure, 0.73 µg/mL, and 0.57 µg/mL, respectively, than cancer cells, where IC_50_ varied from 0.15–0.20 µg/mL, but the selectivity of PG towards cancer cells was not notable, similarly to our results.

Flow cytometric analysis of apoptotic markers in MRC-5 cells revealed the induction of apoptosis triggered by PG and both Br derivatives (Table 3). Since the emission wavelength of prodigiosin was close to that of propidium iodide (PI), only the levels of early apoptosis were detected using Annexin V, and the percentage of necrotic PI positive cells was not evaluated. Exposure of MRC-5 cells to IC_50_ values of PG and both Br derivatives resulted in a comparable level of increase of early Annexin V positive cells compared to the nontreated control (Table 3). Flow cytometry and Annexin V staining were previously used to demonstrate that PG could induce apoptosis of HeLa cells [64].

Number of studies showed a high potential of PG for induction of apoptosis in different cell types. In hematopoietic cancer and brain tumor cells, PG caused apoptotic induction via DNA damage [57], while in HL-60 cells, a model of human promyelocytic leukemia, PG reduces copper ions, which generate ROS and the induction of oxidative copper-mediated DNA cleavage in vitro [65]. Another feature of anticancer activity owing to PG and its complexes with copper and zinc is DNA intercalation and inhibition of topoisomerase I, which can lead to cell-cycle arrest and induce apoptosis [66]. The intracellular target of PG was investigated in melanoma cells, and the apoptotic mechanism of PG was studied in detail, suggesting that PG could interact with BCL-2 family of apoptosis regulatory proteins (BH3 domain of antiapoptotic BLC-2 and BCL-X_L_, MCL-1), which regulate the mitochondrial outer membrane permeabilization [67], and by binding to mTOR (mammalian target of rapamycin) [68], but the interaction between PG and intracellular target proteins needs more precise evaluation, as targets seem diversified [57].

### 2.4. Toxicity Evaluation of Prodigiosin and Its Br Derivatives in C. elegans and D. rerio

PG and its two novel brominated derivatives exhibited survival rate of 100% of *C. elegans* at the highest tested concentration of 50 µg/mL (Table S2 and Figure S7), but it was observed that the motility of nematodes treated with PG-Br_2_ was significantly impaired at all tested concentrations. In line with our findings, toxicological assessment of PG obtained from *S. marcescens* in *C. elegans* showed no significant toxic activity [69], which suggested the safe use of PG in eukaryotic system. Furthermore, PG and its five derivatives, one with an unsaturated hydroxylated chain on C-ring of PG, and four with various cyclic alkyl substituents (C_4_, C_5_, C_6_, and C_10_) on the C-ring, and the structures of derivatives were decisive against the *C. elegans* first stage juveniles [70], with PG exhibiting the highest activity with an EC_50_ value of 0.04 µg/mL, while for the synthetic derivatives of PG, no reliable EC_50_ could be determined, as the mortality at the highest tested concentration did not exceed 33%. These findings, similar to ours, suggest that some synthetic PG derivatives may not prove to be toxic *C. elegans*.

On the other side, the nematocidal activity of PG was reported for juvenile *Meloidogyne incognita* J2 species, harmful on black pepper, with an IC_50_ value of 0.2 mg/mL [71], for juveniles of *Radopholus similis* with LC_50_ 83 µg/mL and *Meloidogyne javanica* with the LC_50_ value of 79 µg/mL, also exhibiting an antagonistic effect on nematode egg-hatching ability [72]. Effectiveness of PG against *Heterodera schachtii* J2 was found to be 4.30 µg/mL [70]. Notably, these values are comparable to IC_50_ (Table 1) which may hinder PG application in the agriculture.

**Figure 5:**
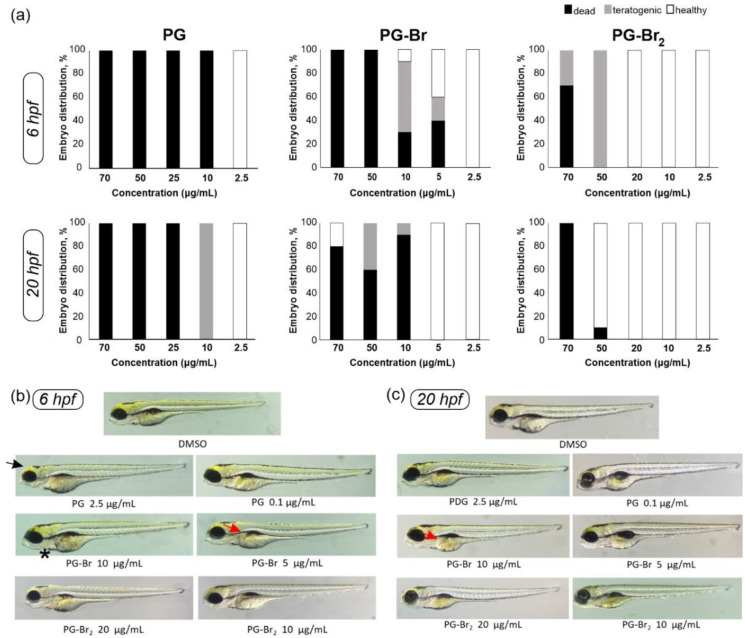
(**a**) Effects on the development of zebrafish embryos treated with different concentrations of PG, PG-Br, and PG-Br_2_, represented as a distribution of alive (white), dead (black), and teratogenic (grey) embryos; zebrafish developed from the embryos: (**b**) 6 hpf and (**c**) 20 hpf, treated with the tested compounds and compared to the DMSO treated control. Black arrow (**→**) points smaller head, red arrow (**→**) points to abnormal liver, and asterisk (*) denotes abnormal heart.

PG, PG-Br, and PG-Br_2_ were assessed for their in vivo toxicity on the vertebrate zebrafish model (Figure 5). Synthetic derivatives of PG have been tested for their activity on fluorescently labeled leukemia cells in vivo on mutant *casper* zebrafish, which lack all pigmentation [73], but the embryotoxicity of PG has not been determined in this model system so far. Compounds were applied at 6- and 20-h post fertilization (hpf), for better insights on the embryotoxicity. Indeed, upon application of 10 µg/mL at 6 hpf, death rate was 100%, 30%, and 0% for PG, PG-Br, and PG-Br_2,_ respectively (Figure 5a). Applying compounds at the 20 hpf, when embryos were in later stages of the development, did result in comparably lower toxicity effects. Nevertheless, PG was found to be the most toxic followed by PG-Br and PG-Br_2_ under both test conditions.

When PG concentrations of 2.5 and 0.1 µg/mL were used, both at 6 hpf and 20 hpf, all the embryos survived, but developed into slightly smaller fish with microcephaly (Figure 5b). At these concentrations, neither of the Br derivatives caused detrimental effects. PG-Br at 5 µg/mL applied at 6 hpf affected 60% of embryos with 20% showing teratogenicity, while this concentration had no effect when applied at 20 hpf (Figure 5a). However, at high concentrations of 50 µg/mL, there was evident uptake of the biopigment inside embryos 6 hpf, and teratogenic effects including hepatotoxicity and cardiotoxicity were noticed in zebrafish 20 hpf (Figure S8a). PG-Br_2_ was the least toxic with the 100% embryos survival rate 6 hpf and 20 hpf at a concentration of 20 µg/mL, without any teratogenic effects (Figure 5a). This concentration is twofold higher in comparison to IC_50_ values (Table 2), indicating that further structural optimizations in terms of halogenation may prove beneficial. Nonetheless, at a higher tested concentration of 50–70 µg/mL of PG-Br_2_, there is evidence of severe toxicity and abnormal development upon application at 6 hpf (Figure S8b).

### 2.5. Drug-Likeness Assessment of Prodigiosin and Its Br Derivatives

Using the methodology known as the Lipinski’s “rule of five”, drug-likeness of PG and Br derivatives was predicted by calculating their physicochemical properties (Table 4), and by assessing their absorption and distribution. “Rule of five” is an effective method that can be applied for the analysis of orally administered drugs, as it applies rules which were associated with 90% of the drugs that have reach clinical phase II trials: MW ≤ 500, log *p* ≤ 5, H–bond donors ≤ 5, H–bond acceptors ≤ 10 [74].

It was noticed that Br derivatives have the partition coefficient (logP) slightly above 5 (Table 4), suggesting a higher hydrophobicity score than PG and less favorable profile for oral availability, thus violating one of Lipinski’s rules. Topological polar surface area (TPSA), a sum of surfaces of polar atoms (usually O and N and their attached hydrogens) predicts drug transport properties, intestinal drug absorption, and blood–brain barrier (BBB) penetration [75], and it remained unchanged for all assessed compounds. Unchanged were the number of HBA (4) and HBD (2), as well as the number of rotatable bonds (7). Indeed, the conformational flexibility of the molecule is described by the number of rotatable bonds and good oral bioavailability is expected for molecules with a maximum of 10 rotatable bonds, where a rotatable bond is defined as any single nonring bond, bounded to a nonterminal, non-hydrogen atom. [76]. Summarizing the physicochemical properties of investigated PGs suggests that they meet the drug-likeness criteria and that further structural modification may help in achieving even better pharmacological properties.

## 3. Materials and Methods

### 3.1. Reagents

All chemicals were of reagent-grade quality or higher and used without further purification; solvents were used as received. Ethyl acetate (EtOAc), hydrogen peroxide (H_2_O_2_), dimethyl sulfoxide (DMSO), HBr, cholesterol, MTT (3-(4,5-Dimethylthiazol-2-yl)-2,5-diphenyltetrazolium bromide), NaHCO_3,_ KH_2_PO_4_, Na_2_HPO_4_, and other salts were purchased from Sigma Aldrich, Steinheim, Germany. Yeast extract, NaCl, and agar were purchased from Biolife, Milan, Italy. Methanol (MeOH), glycerol, Nutrient Broth (NB), and Na_2_SO_4_ were purchased from Fisher Scientific, Loughborough, UK. Tryptone, peptone, Tryptic Soy Broth (TSB) were purchased from Torlak, Belgrade, Serbia. KCl and beef extract were purchased from Becton Dickinson, New Jersey, US. Hexane was purchased from J. T. Baker, Deventer, The Netherlands. MgSO_4_∙7H_2_O was purchased from Acros Organics, Geel, Belgium. Instant Ocean^®^ Salt was purchased from Instant Ocean, Blacksburg, US.

### 3.2. Prodigiosin Production

#### 3.2.1. Bacterial Strain Cultivation

*S. marcescens* ATCC 27117 was purchased from American Type Culture Collection (ATCC, Manassas, VA, USA), kept in 20% glycerol at –80 °C. The producing strain was revived on LA plates (Luria Bertani with 1.5% agar) at 30 °C and grown at 180 rpm (revolutions per minute) at 30 °C overnight in Luria Bertani (LB) for optimization experiments. Five media were assessed for PG production by *S. marcescens* ATCC 27117 (Table S1): LB (Luria Bertani), NB (Nutrient Broth), TSB (Tryptic Soy Broth), SCF (Seed Culture Fluid) and FB (Fermentation Broth) for 5 days at 180 rpm at 30 °C. Cultivation conditions were evaluated for 3 days: pH 4, 5, 6, 7, 8, 9, 10; temperatures: 18, 25, 30, 37 °C; agitation rates: 100, 125, 150, 180, 200 rpm.

#### 3.2.2. Bioreactor Design and Experimental Setup

For the starter culture, *S. marcescens* ATCC 27117 was propagated in NB at 180 rpm at 30 °C for 24 h. Batch cultivations were performed in 3.0 L of the NB production medium with 1% (*v*/*v*) inoculation in the 4.5 L bioreactor (Bio4, EDF-5.4_1, Biotehniskais centrs AS, Riga, Latvia) at 28.0 ± 2.0 °C, pH 7.00 ± 1.00, and maximal 500 rpm agitation rate to secure minimal 40 ± 20% of aeration level during the process for 24 h. The pH was maintained at 7.0 using 10 M NaOH solution and 20% HCl. PG production was monitored spectrophotometrically; 2.5 mL aliquots of the fermentation culture were taken at regular intervals of 1 h (Figure S1), and 1 mL of ethyl acetate (EtOAc) containing 1% HCl was added to 1 mL of bacterial culture, followed by addition of NaCl (ca. 10 mg) to enable phase separation. The phases were separated by 1 min centrifugation at 14,000 rpm in Eppendorf 5418 bench top centrifuge. PG concentration in the organic phase was monitored spectrophotometrically and calculated by measuring the absorbance at the absorption maximum of 535 nm on the spectrophotometer Ultrospec 3300 pro (Amersham Biosciences, Little Chalfont, Amersham, UK) and using the extinction coefficient 51,300 L/(mol × cm) [77]. For the biomass monitoring, 1 mL of the culture was centrifuged for 1 min at 14,000 rpm in Eppendorf 5418 bench top centrifuge, the supernatant was discarded, and the wet cells were dried at 37 °C to a constant weight, which was achieved after 24 h. The fermentation was stopped after 24 h, the cell culture was centrifuged at 6000 rpm for 20 min at 4 °C (Du Pont Instruments Sorvall RC-58 Refrigerated Superspeed Centrifuge, LabX Media Group, Midland, ON, Canada), and bacterial cell pellets were dried at 42 °C to a constant cell dry weight (CDW).

#### 3.2.3. Prodigiosin Extraction and Purification

PG was extracted from dried bacterial cells with methanol (MeOH) containing 1% HCl, and the slurry was disrupted by sonification 3 times for 10 s at 10 mA with 20 s break between sonification (MSE SANYO Soniprep 150 Ultrasonic Disintegrator). Upon centrifugation, bacterial cells were re-extracted twice more using the same protocol. The collected MeOH extract was evaporated under reduced pressure on BÜCHI Rotavapor^®^ R-300 (BÜCHI Labortechnik AG, Flawil, Switzerland) to afford the crude biopigment extract. The crude extract was purified using gravitation column chromatography performed on silica gel (SiO_2_, particle size 0.018–0.032 mm). Solvent mixtures are reported as volume/volume (*v*/*v*). The extract was eluted with n-hexane/Et_2_O 2/1 (150 mL), EtOAc (300 mL), and MeOH (100 mL). Drying under reduced pressure was done at 40 °C on BUCHI Rotavapor^®^ R-300.

### 3.3. Prodigiosin Derivatization

Reactions of PG were monitored by thin-layer chromatography (TLC) carried out on alumina plates with 0.25 mm silica gel layer with F-254 indicator (Kieselgel 60 F_254_, Merck, Darmstadt, Germany) using UV light (254/366 nm) as the visualizing agent.

#### 3.3.1. Monobromination of Prodigiosin

Monobromination of PG was conducted following the literature procedure in [31]. PG (8.0 mg, 0.025 mmol) was dissolved in 1.8 mL MeOH at room temperature; then, 48% HBr in H_2_O (4.17 µL, 0.025 mmol, 1.0 eq) and 30% H_2_O_2_ (3.35 µL, 0.027 mmol, 1.1 eq) were added, and the reaction was allowed to proceed until full conversion. The mixture was evaporated to dryness, water was added, and the product was extracted with EtOAc. The combined organic phases were washed with saturated NaHCO_3_, brine, dried over anhydrous Na_2_SO_4_, filtered, and the solvent was evaporated under reduced pressure. The mixture was purified by gravitation column chromatography on SiO_2_ using n-hexane/EtOAc 9/1 to 8/2. Monobrominated prodigiosin, PG-Br, was obtained as an orange-brown oil (8.12 mg, 0.025 mmol, 81%).

#### 3.3.2. Dibromination of Prodigiosin

Dibromination of PG was conducted following the literature procedure in [56]. PG (9.0 mg, 0.028 mmol) was dissolved in 1.9 mL EtOAc, and 48% HBr in H_2_O (10.38 µL, 0.056 mmol, 2.0 eq) and DMSO (4.37 µL, 0.062 mmol, 2.2 eq) were added. The mixture was heated at 60 °C until full conversion was detected by TLC (2 h). After cooling to room temperature, water was added, and the mixture was extracted with EtOAc. The combined organic phases were washed with saturated NaHCO_3_, brine, dried over anhydrous Na_2_SO_4_, filtered, and the solvent was evaporated under reduced pressure. The mixture was purified by gravitation column chromatography on SiO_2_ using n-hexane/EtOAc 9/1. Dibrominated prodigiosin, PG-Br_2_, was obtained as a purple film (8.5 mg, 0.018 mmol, 63%).

#### 3.3.3. Structural Characterization of Prodigiosin and its Br derivatives

Ultraviolet spectra (UV) were recorded on the Ultrospec 3300 pro (Amersham Biosciences, Little Chalfont, Amersham, UK). The structures of PG and its Br derivatives were confirmed by ^1^H- and ^13^C-NMR spectroscopy with the Bruker Avance III spectrometer 500 and 126 MHz, respectively, at room temperature. The compounds were soluble in methanol-d_4_ (Carl Roth GMBH, Karlsruhe, Germany) or deuterated-chloroform (CDCl_3_, Acros Organics, Darmstadt, Germany) and transferred into a 5 mm NMR tube. Chemical shifts (δ) are expressed in ppm and coupling constants (J) are given in Hz. Mass spectra were determined by HPLC-MS (Thermo LCQ fleet coupled with a Dionex UltiMate 3000 HPLC system, Horsham, UK) and by HR-LC-ESI-MS (Thermo LTQ FT Ultra coupled with a Dionex UltiMate 3000 HPLC system, Loughborough, UK) in a positive ion polarity mode.

### 3.4. Biological Assays

#### 3.4.1. Cytotoxicity and Flow Cytometry Analysis

Selected cell lines MRC-5 (lung fibroblasts), A549 (lung cancer), A375 (melanoma, skin cancer), MDA-MB-231 (breast cancer), and HCT116 (colon cancer) were obtained from ATCC, Manassas, US. Antiproliferative activities of PG and its Br derivatives were measured using the standard colorimetric MTT [3-(4,5-dimethylthiazol-2-yl)-2,5-diphenyltetrazolium bromide] assay [78]. Briefly, cells were plated in a 96-well flat-bottom plate (Sarstedt, Nümbrecht, Germany) at a concentration of 1 × 10^4^ cells per well, grown in a humidified atmosphere of 95% air and 5% CO_2_ at 37 °C, and maintained as monolayer cultures in RPMI 1640 medium supplemented with 10% fetal bovine serum (FBS), 100 U/mL penicillin, and 100 μg/mL streptomycin (all media components from Gibco™ by Thermo Fischer Scientific CE). Each tested compound was added to the cells at a concentration of 0.05–100.00 μg/mL, and the treatment lasted for 48 h. The MTT assay was performed two times in four replicates, and the results were presented as a percentage of the DMSO-treated control that was arbitrarily set to 100%. Plates were read using the Tecan Infinite 200 Pro multiplate reader (Tecan Group Ltd., Männendorf, Switzerland).

To assess the effect of the treatment duration, exposure of MRC-5 and HCT116 cells to PG and its Br derivatives was observed after 24 h, 48 h, and 72 h and measured using the MTT assay, as described above. MRC-5 and HCT-116 cells treated for 24 h were additionally analyzed using DM IL LED Inverted Microscope (Leica Microsystems, Mannheim, Germany) at 20× magnification.

The eBioscience^TM^ Annexin V Apoptosis Detection Kit APC (Invitrogen by Thermo Fischer Scientific, Waltham, MA, USA) was used for the assessment of cellular integrity and the externalization of phosphatidylserine. MRC-5 cells were plated in a 6-well flat-bottom plate at a concentration of 2.5 × 10^5^ cells per well, grown for 24 h in a humidified atmosphere of 95% air and 5% CO_2_ at 37 °C in RPMI 1640 medium supplemented with 10% fetal bovine serum (FBS), 100 U/mL penicillin, and 100 μg/mL streptomycin (Gibco™ by Thermo Fischer Scientific CE, Waltham, MA, USA). Each tested compound was added to the cells at IC_50_ concentration, and the incubation continued for another 24 h under the same conditions. Upon incubation, cells were collected, washed once with Dulbecco’s Phosphate-Buffered Saline (Gibco™ by Thermo Fischer Scientific CE, Waltham, MA, USA), and stained with Annexin V FITC and propidium iodide (PI) under the manufacturer’s instructions. Briefly, MRC-5 cells were resuspended in 1 × Binding Buffer at 1–5 × 10^6^ cells per mL and incubated with 5.00 µL Annexin V FITC for 15 min at room temperature in a final volume of 100 μL. After incubation, cells were washed and resuspend in 200 μL of 1 × Binding Buffer, 5.00 μL of Propidium Iodide Staining Solution was added, and incubation at room temperature continued for another 15 min. Immediately after staining, cells were analyzed using a CyFlow Space Partec flow cytometer, with Partec FloMax software (Partec GmbH, Münster, Germany).

#### 3.4.2. Roundworm (*C. elegans*) Survival Assay

Nematode roundworms (*Caenorhabditis elegans* AU37) were obtained from the Caenorhabditis Genetics Center (CGC), University of Minnesota, Minneapolis, Minnesota, US. *C. elegans* AU37 (glp-4; sek-1) was propagated under standard conditions, synchronized by hypochlorite bleaching, and cultured on nematode growth medium using *E. coli* OP50 as a food source, as described previously [79]. The *C. elegans* survival assay was carried out following the standard procedure with some modifications [80]. Briefly, synchronized worms (L4 stage) were suspended in a medium containing 95% M9 buffer (3.0 g of KH_2_PO_4_, 6.0 g of Na_2_HPO_4_, 5.0 g of NaCl, and 1 mL of 1 M MgSO_4_∙7H_2_O in 1 L of water), 5% LB broth, and 10 μg/mL of cholesterol (Sigma-Aldrich, Munich, Germany). The experiment was carried out in 96-well flat-bottomed microtiter plates (Sarstedt, Nümbrecht, Germany) in the final volume of 100 μL per well. 25 μL of this suspension of nematodes (25–35 nematodes) were transferred to the wells of a 96-well microtiter plate, where 50 μL of the medium was previously added. Next, 25 μL of the solvent control (1% DMSO) or 25 μL of the concentrated tested compound solution was added to the test wells. Final concentrations of the compounds were 50.00, 25.00, 12.50, 6.25, 3.13, and 1.57 µg/mL. Subsequently, the plates were incubated at 25 °C for 2 days. The fraction of dead worms was determined after 48 h by counting the number of dead worms and the total number of worms in each well, using a Carl Zeiss™ Stemi 508 Stereomicroscope (Zeiss Group, Jena, Germany) at 40× magnification. The compounds were tested three times in each assay and each assay was repeated two times (*n* = 6). As a negative control experiment, nematodes were exposed to the medium containing 1% DMSO.

#### 3.4.3. Zebrafish (*D*. *rerio*) Embryotoxicity

Wild-type zebrafish (*Danio rerio*) strain was obtained from the commercial supplier Pet Center, Belgrade, Serbia. Zebrafish embryotoxicity assay was performed according to general rules of the OECD Guidelines for the testing of chemicals [81]. The wild-type zebrafish (*D. rerio*) strain was kept under controlled environmental conditions (water temperature 28 °C, 14 h under the light and 10 h in the dark) and fed regularly three times a day with commercially dry flake food supplemented with *Artemia nauplii* (TetraMinTM flakes; Tetra Melle, Germany). Zebrafish embryos were produced by female and male adults mating in the ratio of 1:2. Obtained embryos were collected and washed from detritus and carefully handled. Only fertilized embryos were selected and distributed into 24-well plates (Sarstedt, Germany) containing 10 embryos per well. Each well contained exactly 1 mL of water for embryos (0.2 g/L of Instant Ocean^®^ Salt in distilled water). For assessing lethal and developmental toxicity, embryos at the 6 and 20 h postfertilization (hpf) stages [82] were treated with selected compounds at concentrations of 0.1–100 μg/mL (maximum DMSO concentration in the negative control was 1%) and incubated at 28 °C. Experiments were performed in triplicate, using 30 embryos for each concentration. For five days, the appearance of different morphophysiological parameters in embryo development was monitored [83,84], and dead embryos were counted and discarded every 24 h. On the fifth day, the embryos were anesthetized by the addition of 0.1% (*w*/*v*) tricaine solution (Sigma-Aldrich, St. Louis, Missouri, US), observed under a stereomicroscope (SMZ-143-N2GG, Motic, Germany) at 3.5× magnification, photographed, and killed by freezing at −20 °C for ≥24 h. All experiments involving zebrafish were performed in compliance with the European directive 2010/63/EU and the ethical guidelines of the Guide for Care and Use of Laboratory Animals of the Institute of Molecular Genetics and Genetic Engineering, University of Belgrade, Serbia.

### 3.5. Drug-Likeness Calculations

Molinspiration tool (©Molinspiration Cheminformatics 2022, free web services, available online: https://www.molinspiration.com, accessed on 26 May 2022, Slovensky Grob, Slovakia) was used for calculating physicochemical properties of the investigated compounds.

## 4. Conclusions

Low yield and high cost of extraction and purification still represent the bottleneck in microbial production of prodigiosin. In this study, we report straightforward fermentation of *S. marcescens* ATCC 27117 in standard Nutrient Broth, purification, and generation of novel halogenated (brominated) derivatives of PG. Although the biological activity of PG in the producing organism is not fully understood, it shows numerous biological functions in other organisms, including anticancer properties. Br derivatives of PG retained anticancer properties and showed favorable toxicity profiles in vivo, with satisfying pharmacokinetic profiles and drug-likeness properties.

## Figures and Tables

**Table 1:** Antiproliferative activity of PG, PG-Br, and PG-Br_2_ (IC_50_, µg/mL) after 48 h treatment.

	IC_50_ (µg/mL)		
Cell Line	PG	PG-Br	PG-Br_2_
MRC-5	1.20 ± 0.04	5.50 ± 0.02	10.00 ± 0.09
A549	1.30 ± 0.02	8.00 ± 0.06	16.00 ± 0.08
A375	1.25 ± 0.04	6.00 ± 0.05	12.00 ± 0.04
MDA-MB-231	0.62 ± 0.01	6.25 ± 0.04	17.00 ± 0.05
HCT116	0.70 ± 0.02	5.00 ± 0.05	10.00 ± 0.08

**Table 2:** Effect of exposure time on cytotoxicity of PG, PG-Br, and PG-Br_2_ using MRC-5 and HCT116 cell lines.

Exposure Time (h)	IC_50_ (µg/mL)		
	PG	PG-Br	PG-Br_2_
Cell line MRC-5			
24	1.80 ± 0.04	7.60 ± 0.08	15.00 ± 0.05
48	1.20 ± 0.04	5.50 ± 0.02	10.00 ± 0.09
72	0.70 ± 0.02	5.98 ± 0.09	10.00 ± 0.08
Cell line HCT116			
24	5.00 ± 0.06	35.00 ± 0.09	50.00 ± 0.09
48	0.70 ± 0.02	5.00 ± 0.05	10.00 ± 0.08
72	0.80 ± 0.02	3.20 ± 0.06	10.00 ± 0.05

**Table 3:** Early apoptosis induction in MRC-5 cell line by PG, PG-Br, and PG-Br_2_.

Annexin V Positive Cells	PG	PG-Br	PG-Br_2_
(%)	20.3 ± 0.5	21.3 ± 0.8	19.6 ± 0.6

**Table 4:** Calculated physicochemical properties of the investigated PGs for the assessment of drug-likeness.

Comp.	miLogP ^a^	TPSA ^b^	N_atoms_ ^c^	MW ^d^	N_ON_ ^e^	N_OHNH_ ^f^	N_viol._ ^g^	N_rotb._ ^h^	Vol ^i^
PG	4.70	53.71	24	323.44	4	2	0	7	315.55
PG-Br_2_	6.17	53.71	26	481.23	4	2	1	7	354.32
PG-*2*-Br	5.63	53.71	25	402.34	4	2	1	7	336.44
PG-*3*-Br	5.43	53.71	25	402.34	4	2	1	7	336.44
PG-*4*-Br	5.43	53.71	25	402.34	4	2	1	7	336.44
PG-*3′*-Br	5.43	53.71	25	402.34	4	2	1	7	336.44
PG-*6′*-Br	5.49	53.71	25	402.34	4	2	1	7	336.44
PG-*3″*-Br	5.43	53.71	25	402.34	4	2	1	7	336.44

“Rule of five” ≤ 5; ^a^ Octanol–water partition coefficient; ^b^ topological polar surface area (Å^2^); ^c^ number of nonhydrogen atoms; ^d^ molecular weight; ^e^ number of hydrogen-bond acceptors (HBA, O, and N atoms); ^f^ number of hydrogen-bond donors (HBD, OH, and NH groups); ^g^ number of “Rule of five” violations; ^h^ number of rotatable bonds; ^i^ molecular volume (Å^3^).

## Data Availability

The data presented in this study are available on request from the corresponding author.

## Supplementary Materials

The following are available online at https://www.mdpi.com/article/10.3390/molecules27123729/s1, Figure S1: title, **Table S1:** The media composition for the nutritional factors’ optimization for *S. marcescens* ATCC 27117 cultivation; **Figure S1**: Fermentation of *S. marcescens* ATCC 27117 in NB, samples taken every 1 h for 24 h, PG production (primary *y*-axis) and cell dry weight (secondary *y*-axis) were monitored; **Figure S2:** ^1^H- and ^13^C-NMR spectra of bacterial prodigiosin (PG); **Figure S3.** Chemical monobromination reaction of PG: (**a**) HPLC chromatogram for monobromination; (**b**) *m/z* 402.25 and 404.25 (1:1) at RT = 10.18 min; **Figure S4:** ^1^H-NMR spectrum of PG-Br isomers (approximate ratio 1:1); **Figure S5:** ^1^H- and ^13^C-NMR spectra of PG-Br_2_; **Figure** **S6**: (**a**–**d**) MRC-5 and (**e**–**h**) HCT116 cells after 24 h treatment with PG and Br derivatives. Images were taken under DM IL LED Inverted Microscope (Leica Microsystems, Germany) at 20× magnification; **Table S2:** Influence of PG and its novel Br derivatives on the survival rate of juvenile *C. elegans*; **Figure S7**: In vivo toxicity of PG and Br derivatives using *C. elegans* model system. Nematodes were treated with 50 µg/mL of: (**a**) PG; (**b**) PG-Br; (**c**) PG-Br_2_; (**d**) DMSO. Images were taken under Stereomicroscope Carl Zeiss™ Stemi 508 (Germany) at 40× magnification; **Figure S8**: Effects on zebrafish from the treatment of higher concentrations of 50 µg/mL of: (**a**) PG-Br; (**b**) PG-Br_2_, compared to (**c**) DMSO control. Images were taken under a stereomicroscope (SMZ143-N2GG, Motic, Germany) at 3.5× magnification. Red arrow (→) points to abnormal liver and asterisk (*) denotes abnormal heart.
