# Supplementary material for: Synthesis, Anticancer Potential and Comprehensive Toxicity Studies of Novel Brominated Derivatives of Bacterial Biopigment Prodigiosin from Serratia marcescens ATCC 27117
Source: Molecules. 2022 Jun 9;27(12):3729. doi: 10.3390/molecules27123729 (PMC9227013; doi:10.3390/molecules27123729)
Supplement: Supplementary file 1 [file molecules-27-03729-s001.zip › molecules-1739929-supplementary.pdf]

Synthesis, Anticancer Potential and Comprehensive Toxicity Studies of Novel Brominated Derivatives of Bacterial Biopigment Prodigiosin from *Serratia marcescens* ATCC 27117

Jelena Lazic [1,2], Sanja Skaro-Bogojevic [2], Sandra Vojnovic [2], Ivana Aleksic [2], Dusan Milivojevic [2], Martin Kretzschmar [3], Tanja Gulder [3], Milos Petkovic [4] and Jasmina Nikodinovic-Runic [2,*]

[1] Graduate School, University of Nova Gorica, SI-5000 Nova Gorica, Slovenia; jelena.lazic@student.ung.si
[2] Institute of Molecular Genetics and Genetic Engineering, University of Belgrade, 11000 Belgrade, Serbia; sanja.bogojevic@imgge.bg.ac.rs (S.S.B.); sandravojnovic@imgge.bg.ac.rs (S.V.); ivana_aleksic@imgge.bg.ac.rs (I.A.); dusan.milivojevic@imgge.bg.ac.rs (D.M.)
[3] Institute of Organic Chemistry, Leipzig University, 04103 Leipzig, Germany; tanja.gulder@uni-leipzig.de (T.G.); martin.kretzschmar@uni-leipzig.de (M.K.)
[4] Faculty of Pharmacy, University of Belgrade, 11000 Belgrade, Serbia; milosp@pharmacy.bg.ac.rs (M.P.)
* Correspondence: jasmina.nikodinovic@imgge.bg.ac.rs (J.N.R.)

Supplementary Materials

Table of contents

Table S1. The media composition for the nutritional factors' optimization 1

Figure S1. Fermentation of *S. marcescens* ATCC 27117 in NB 1

Figure S2. ^{1}H- and ^{13}C-NMR spectra of bacterial prodigiosin 1-2

Figure S3. Analysis of the HPLC-MS spectrum of PG-Br reaction mixture 3

Figure S4. ^{1}H-NMR spectrum of PG-Br isomers 4

Figure S5. ^{1}H- and ^{13}C-NMR spectra of PG-Br_2 5-6

Figure S6. Microscopy results 7

Table S2. and **Figure S7.** Survival rate of *C. elegans* 8

Figure S8. Zebrafish embryotoxicity 8

Table S1. The media composition for the nutritional factors' optimization for *S. marcescens* ATCC 27117 cultivation.

Medium[a]	Composition
LB	10.0 g/L tryptone, 5.0 g/L yeast extract, 10.0 g/L NaCl
NB	5.0 g/L peptone, 1.0 g/L beef extract, 2.0 g/l yeast extract, 5.0 g/L NaCl
TSB	30.0 g/L Tryptic Soy Broth
SCF	10.0 g/L peptone, 5.0 g/L yeast extract, 3.0 g/L NaCl, 2.0 g/L KCl
FB	15.0 g/L peptone, 0.3% (v/v) glycerol, 3.0 g/L NaCl, 2.0 g/L KCl, 2.0 g/L $MgSO_4$

[a]Abbreviations: **LB** (Luria Bertani), **NB** (Nutrient Broth), **TSB** (Tryptic Soy Broth), **SCF** (Seed Culture Fluid), **FB** (Fermentation Broth)

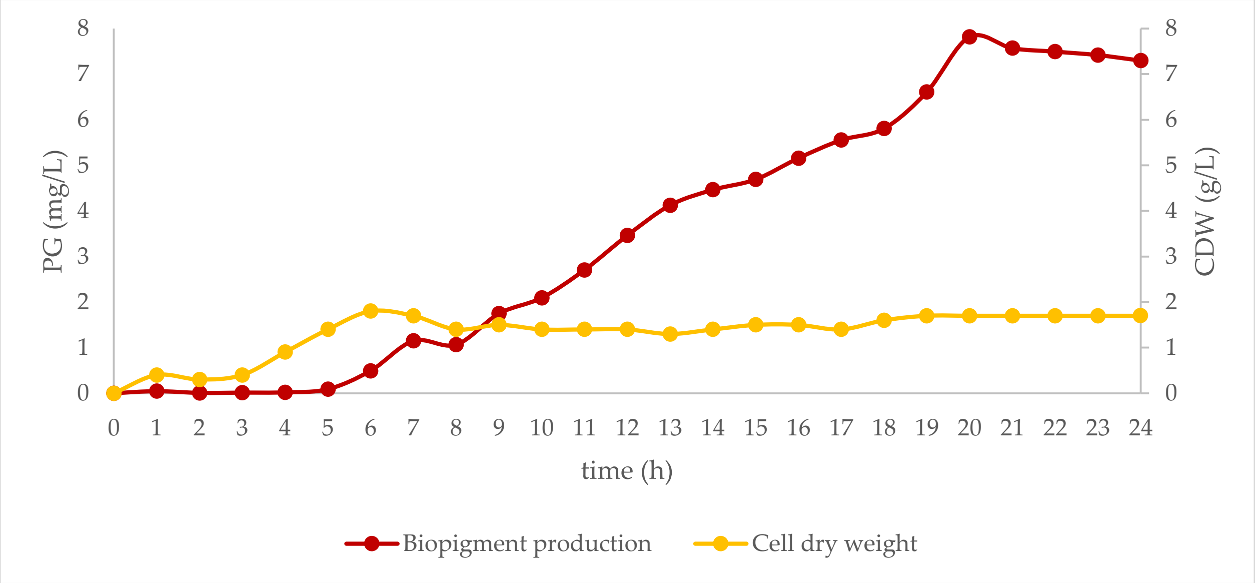

Figure S1. Fermentation of *S. marcescens* ATCC 27117 in NB, samples taken every 1 h for 24 h, PG production (primary *y*-axis) and cell dry weight (secondary *y*-axis) were monitored.

Characterization of Prodigiosin

4-Methoxy-5-[(5-methyl-4-pentyl-2*H*-pyrrol-2-ylidene)methyl]-1*H*,1'*H*-2,2'-bipyrrole, $C_{20}H_{25}N_3O$, PG, was obtained as a red film (21 mg, 0.065 mmol). UV: λ_{max} (EtOAc, nm) 535, 501sh.^{1}H-NMR (500 MHz, methanol-*d₄*) δ 7.23 (s, H-6′), 7.18 (d, *J* = 2.6 Hz, H-2), 7.05 – 7.03 (m, H-3′′ and H-4), 6.45 (s, H-3′), 6.39 (dd, *J* = 3.7, 2.6 Hz, H-3), 4.04 (s, 3H, –OMe), 2.45 – 2.41 (m, 2H, H-7′′), 2.36 (s, 3H, H-6′′), 1.61 – 1.57 (m, 2H, H-8′′), 1.39 – 1.35 (m, 4H, H- 9′′ and H-10′′), 0.94 – 0.91 (m, 3H, H-11′′). ^{13}C-NMR (126 MHz, methanol-*d4*) δ 168.5 (C-5′′), 151.1 (C-2′′), 149.1 (C-3′′), 127.6 (C-4′′), 127.2 (C-2′), 123.9 (C-5), 123.3 (C-2), 117.7 (C-5′), 117.2 (C-6′), 115.0 (C-4), 113.1 (C-4′), 113.0 (C-3), 94.9 (C-3′), 59.8 (–OMe), 32.8 (C-9′′), 31.3 (C-7′′), 30.8 (C-8′′), 23.6 (C-10′′), 14.5 (C-6′′), 12.0 (C-11′′) ppm. HR-LC-ESI-MS: *m*/*z* calculated for $C_{20}H_{25}N_3O^+$ [M+H]$^+$ 324.1998 (100.0%), found 324.2098 (100.0%).

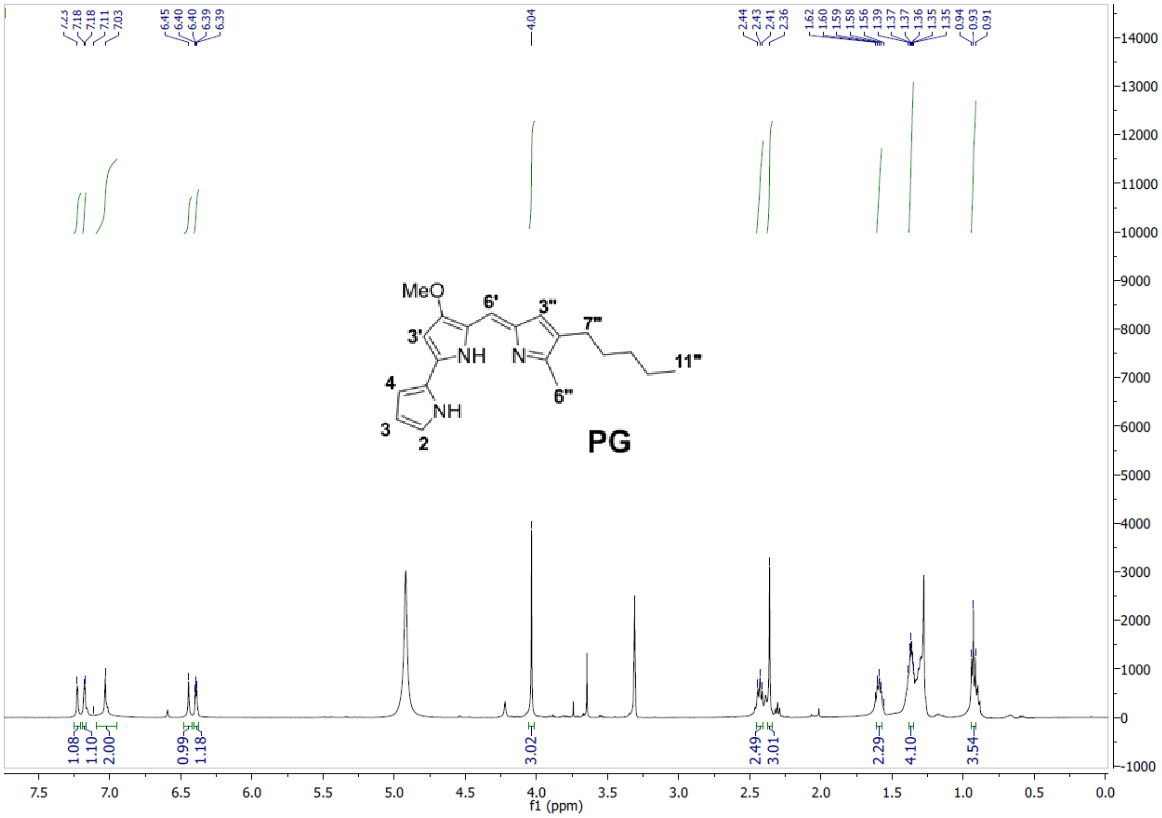

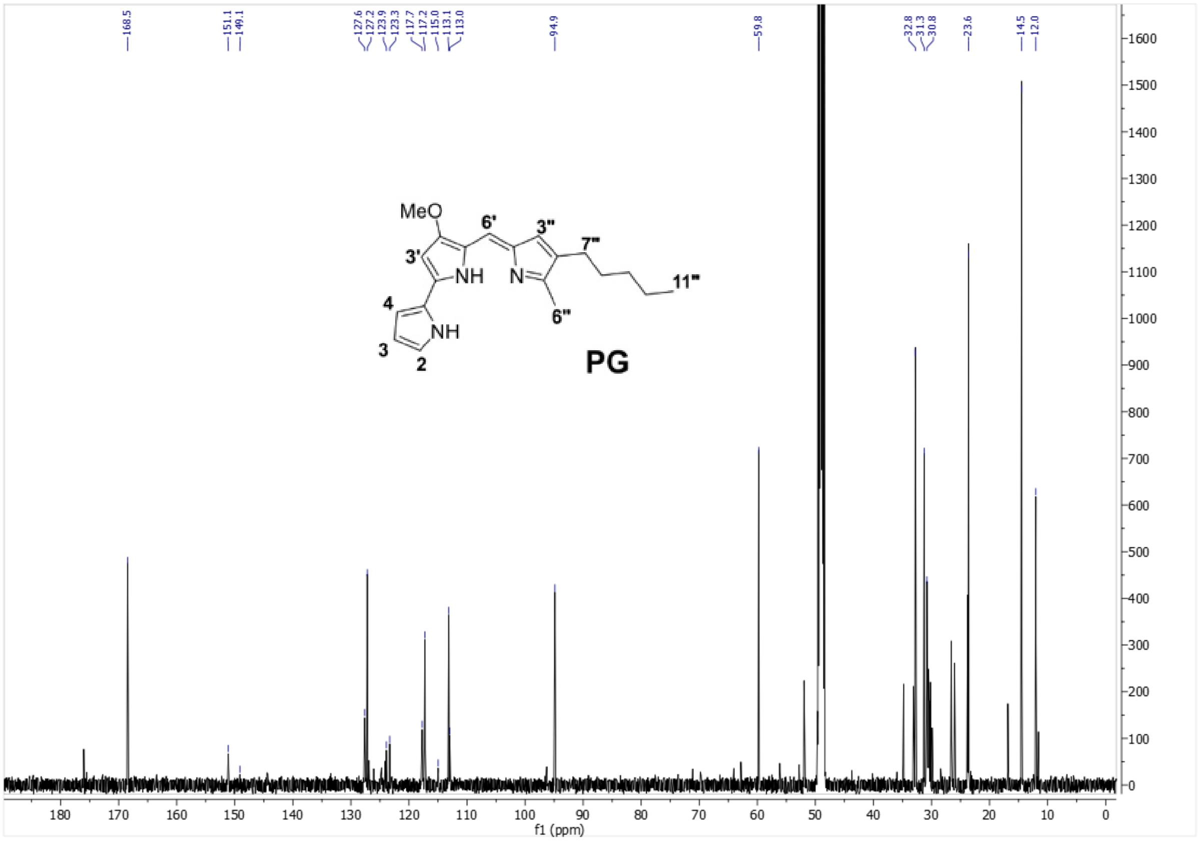

Figure S2. ^{1}H- and ^{13}C-NMR spectra of bacterial prodigiosin (PG).

Characterization of (Assumed) Monobrominated Prodigiosins

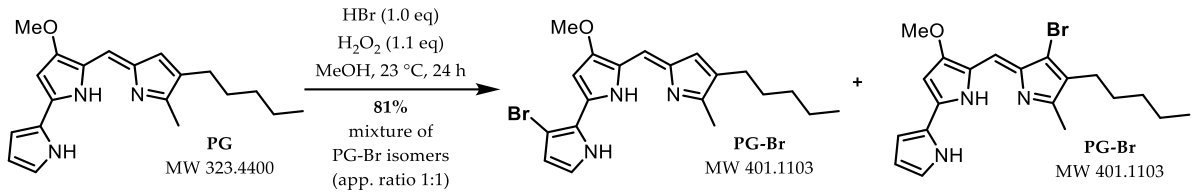

The crude reaction mixture of monobromination of PG was monitored by HPLC-MS and the most abundant peak in the chromatogram had the expected $[M+H]^+$ signal for PG-Br, with *m/z* 402.25 and 404.25, with signal intensities 1:1, characteristic for monobrominated compounds (Figure S3), while the *m/z* of PG was not found.

(a) RT (retention time) = 0.00 – 25.99 min (b) RT = 10.18 min

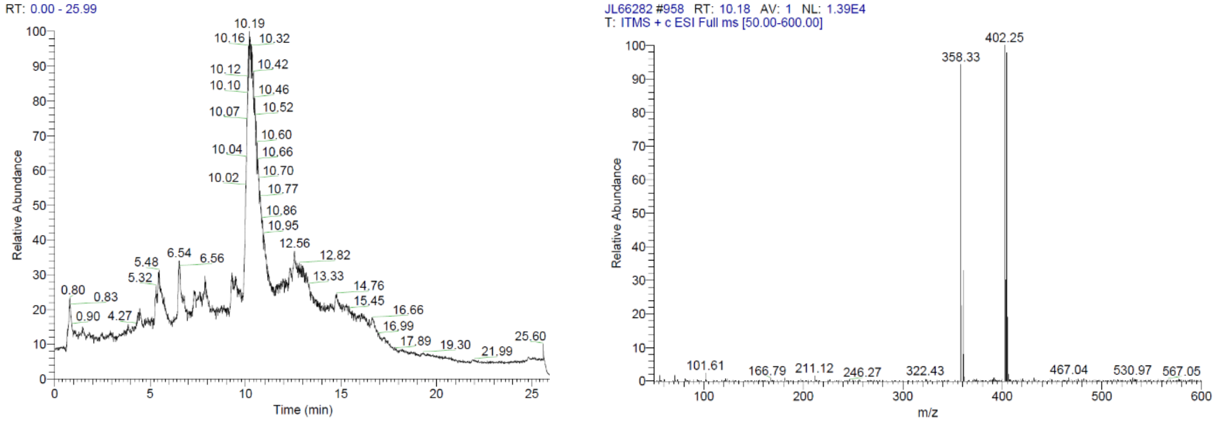

Figure S3. Chemical monobromination reaction of PG: (**a**) HPLC chromatogram for monobromination; (**b**) *m/z* 402.25 and 404.25 (1:1) at RT = 10.18 min.

Upon reaction completion, PG-Br was worked-up and purified as described in the Section 3.3.1 in the main text. PG-Br characterisation is provided below.

Bromo-4'-methoxy-5'-[(5-methyl-4-pentyl-2H-pyrrol-2-ylidene)methyl]-1*H*,1'*H*-2,2'-bipyrrole, $C_{20}H_{24}BrN_3O$, PG-Br, was obtained as an orange-brown oil (8.12 mg, 0.025 mmol, 81%). UV/Vis: λ_{max} (EtOAc, nm) 540, 470. ^1H NMR (500 MHz, methanol-d_4) δ 7.73 – 7.71 (m, 1H), 7.64 – 7.59 (m, 1H), 7.47 – 7.45 (m, 2H), 7.33 – 7.25 (m, 3H), 7.23 – 7.22 (m, 1H), 7.22 – 7.21 (m, 1H), 7.00 – 6.95 (m, 1H), 4.11 (s, 3H), 4.09 (s, 3H), 2.64 – 2.55 (m, 3H), 2.51 – 2.49 (m, 2H), 2.48 – 2.46 (m, 2H), 2.47 – 2.45 (m, 2H), 2.43 – 2.41 (m, 2H), 2.41 – 2.39 (m, 2H), 2.39 – 2.37 (m, 2H), 2.37 – 2.35 (s, 2H), 2.28 – 2.24 (m, 3H), 1.76 – 1.72 (m, 2H), 1.65 – 1.63 (m, 6H). The signals in the ^{13}C-NMR spectrum was not legible due to overlapping of signals from different isomers, as well as the residual solvent signals. Therefore, the 2D spectra (HSQC, HMBC) were not legible and are not presented. HR-LC-ESI-MS: *m/z* calculated for $C_{20}H_{25}BrN_3O^+$ $[M+H]^+$ 402.1176 (100.0%), 404.1155 (97.3%); found 402.1157 (100.0%), 404.1141 (99.4%).

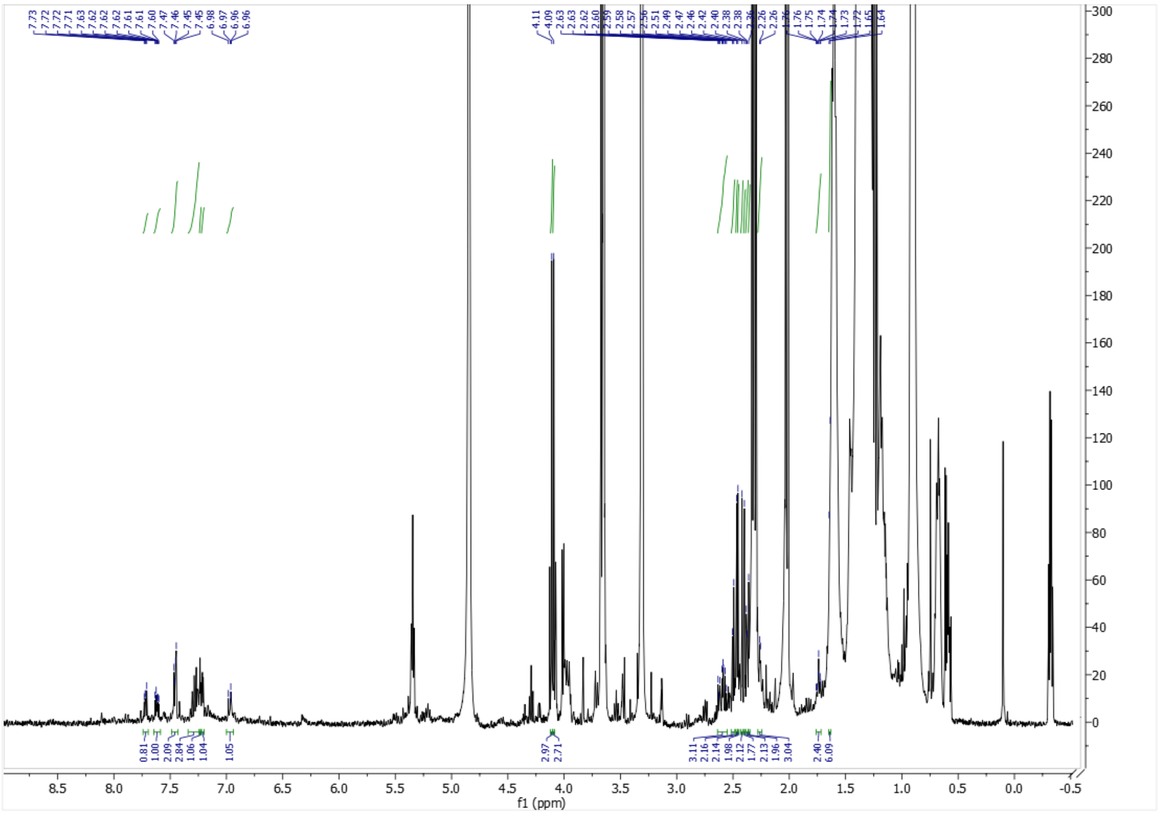

Figure S4. ^{1}H-NMR spectrum of PG-Br isomers (approximate ratio 1:1).

Characterization of Dibrominated Prodigiosin

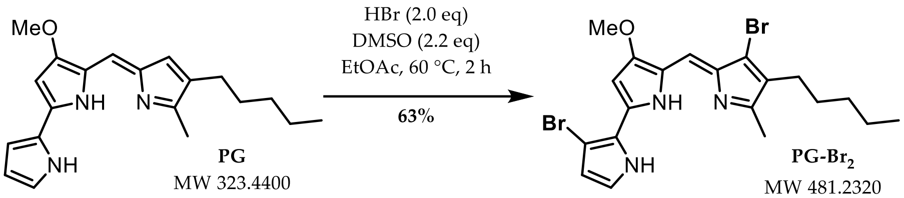

The crude reaction mixture of dibromination of PG was monitored by HPLC-MS and the most abundant peak in the chromatogram had the expected $[M+H]^+$ signal for PG-Br_2, with *m/z* 480.21, 482.20, 484.20, with signal intensities 1:2:1, characteristic for the dibrominated compounds, while the *m/z* of PG was not found. Upon reaction completion, PG-Br_2 was worked-up and purified as described in the Section 3.3.2 in the main text. PG-Br_2 characterisation is provided below.

3-Bromo-5'-[(3-bromo-5-methyl-4-pentyl-2H-pyrrol-2-ylidene)methyl]-4'-methoxy-1*H*,1'*H*-2,2'-bipyrrole, $C_{20}H_{23}Br_2N_3O$, PG-Br_2, was obtained as a purple film (8.5 mg, 0,018 mmol, 63%). UV/Vis: λ_{max} (EtOAc, nm) 544, 480. ^{1}H-NMR (500 MHz, $CDCl_3$) δ 7.24 (s, H-6′), 7.18 (d, *J* = 4.0 Hz, H-2), 6.55 (s, H-3′), 6.44 (d, *J* = 4.0 Hz, H-3), 4.09 (s, 3H, –OMe), 2.53 – 2.50 (m, 2H, H-7′′), 2.40 (s, 3H, H-6′′), 1.65 – 1.62 (m, 2H, H-8′′), 1.40 – 1.37 (m, 4H, H- 9′′ and H-10′′), 0.95 – 0.87 (m, 3H, H-11′′). ^{13}C-NMR (126 MHz, $CDCl_3$) δ 168.6 (C-5′′), 148.8 (C-2′′), 127.7 (C-4′′), 124.9 (C-2′), 123.4 (C-5), 121.1 (C-3′′), 118.6 (C-6′), 118.2 (C-5′), 118.1 (C-2), 115.7 (C-4′), 110.0 (C-3), 104.8 (C-4), 94.9 (C-3′), 59.9 (–OMe), 32.8 (C-9′′), 31.3 (C-7′′), 30.8 (C-8′′), 23.6 (C-10′′), 14.4 (C-6′′), 12.1 (C-11′′) ppm. HR-LC-ESI-MS: *m/z* calculated for $C_{20}H_{24}BrN_3O^+$ $[M+H]^+$ 480.0281 (51.4%), 482.0260 (100.0%), 484.0240 (48.6%); found 480.0256 (50.2%), 482.0239 (100.0%), 484.0223 (49.7%).

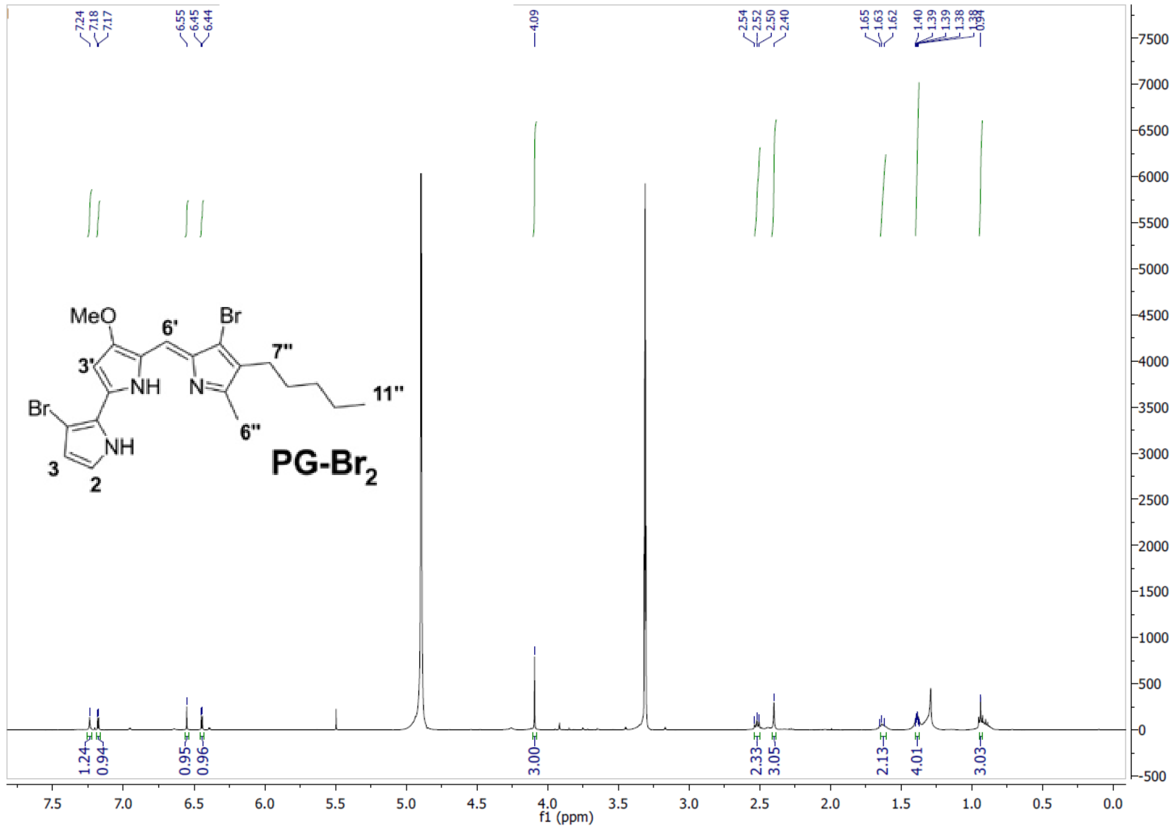

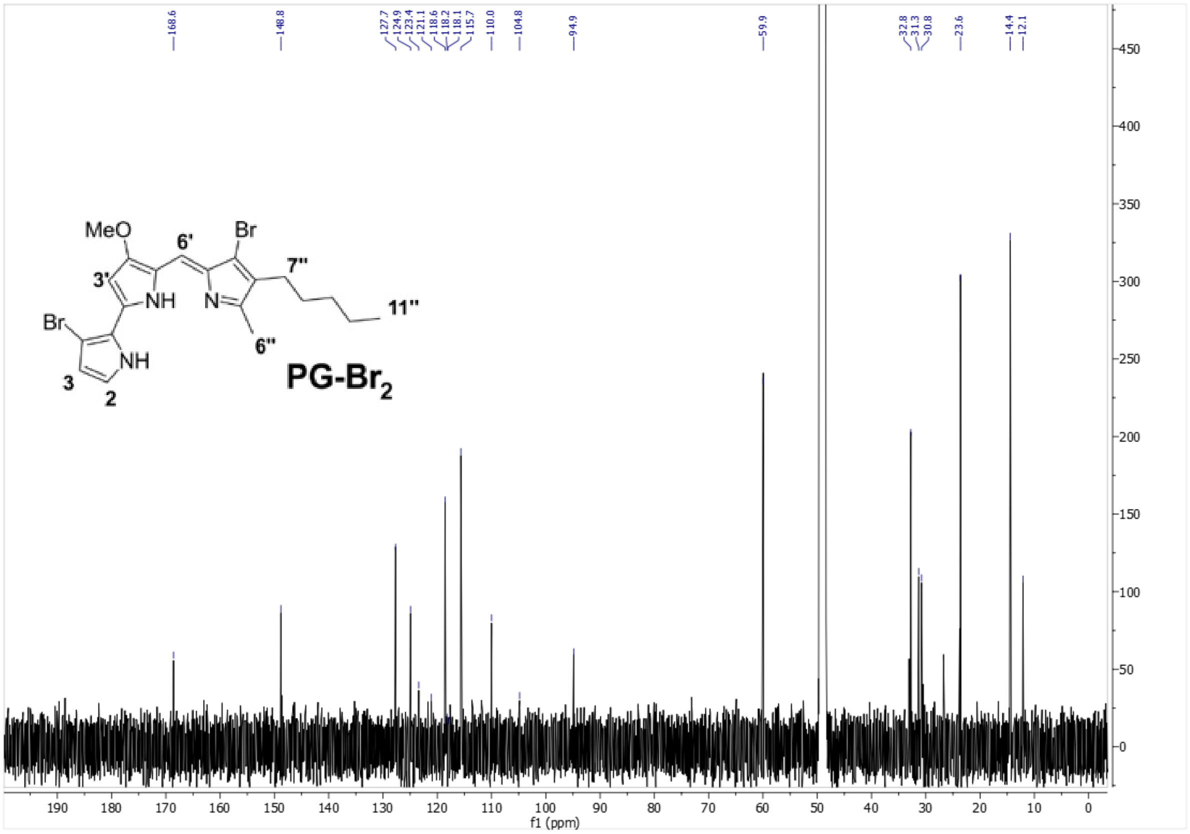

Figure S5. ^{1}H- and ^{13}C-NMR spectra of PG-Br_2.

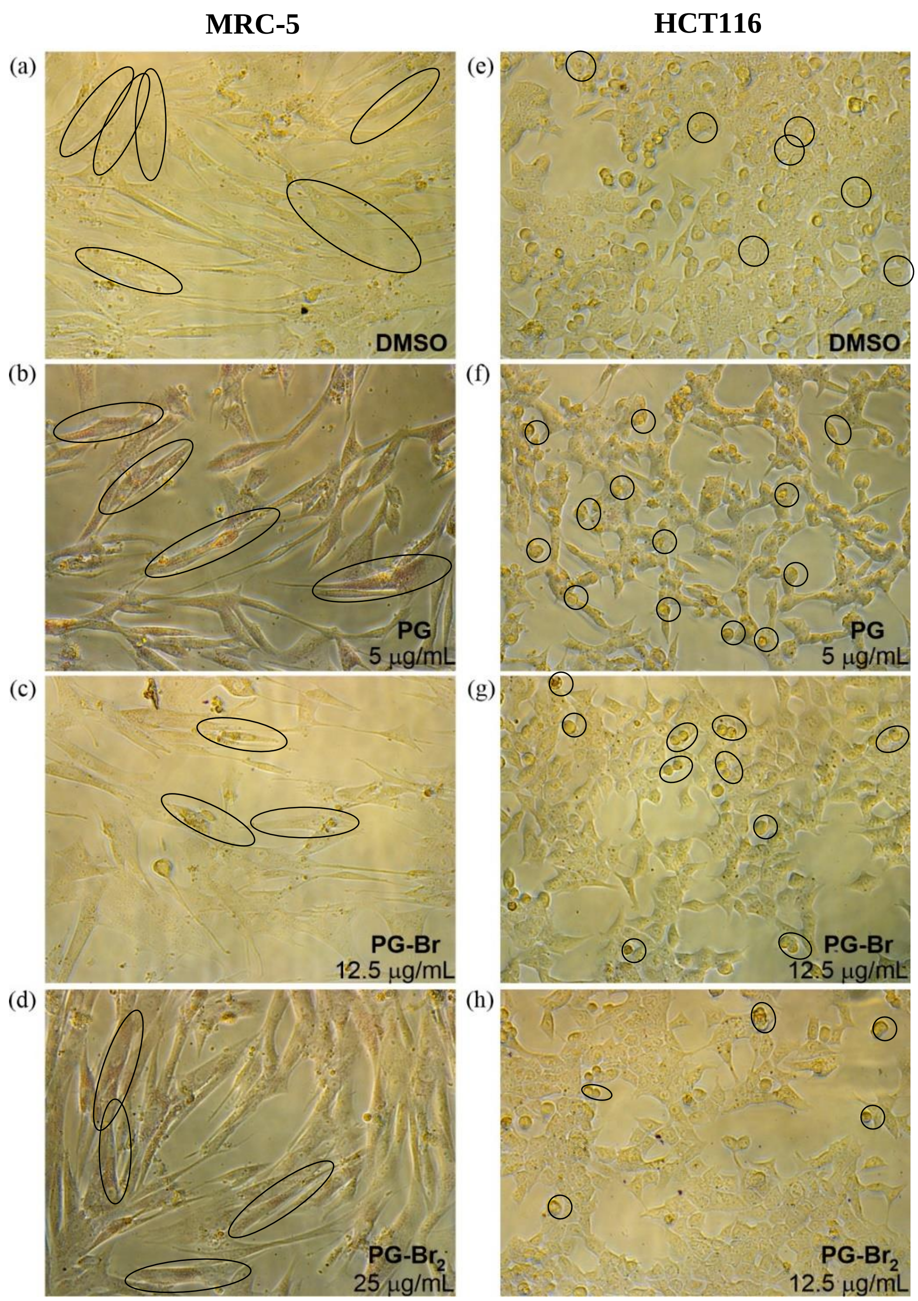

Figure S6. (a-d) MRC-5 and (e-h) HCT116 cells after 24 h treatment with PG and Br-derivatives. Images were taken under DM IL LED Inverted Microscope (Leica Microsystems, Germany) at 20× magnification.

Table S2. Influence of PG and its novel Br-derivatives on the survival rate of juvenile *C. elegans*.

Concentration (μg/mL)	Survival rate (%) PG	PG-Br	$PG-Br_2$
50.00	100 ± 0.00	100 ± 0.00	100 ± 0.00
25.00	100 ± 0.00	100 ± 0.00	100 ± 0.00
12.50	100 ± 0.00	100 ± 0.00	100 ± 0.00
6.25	100 ± 0.00	100 ± 0.00	100 ± 0.00
3.13	100 ± 0.00	100 ± 0.00	100 ± 0.00
1.57	100 ± 0.00	100 ± 0.00	100 ± 0.00

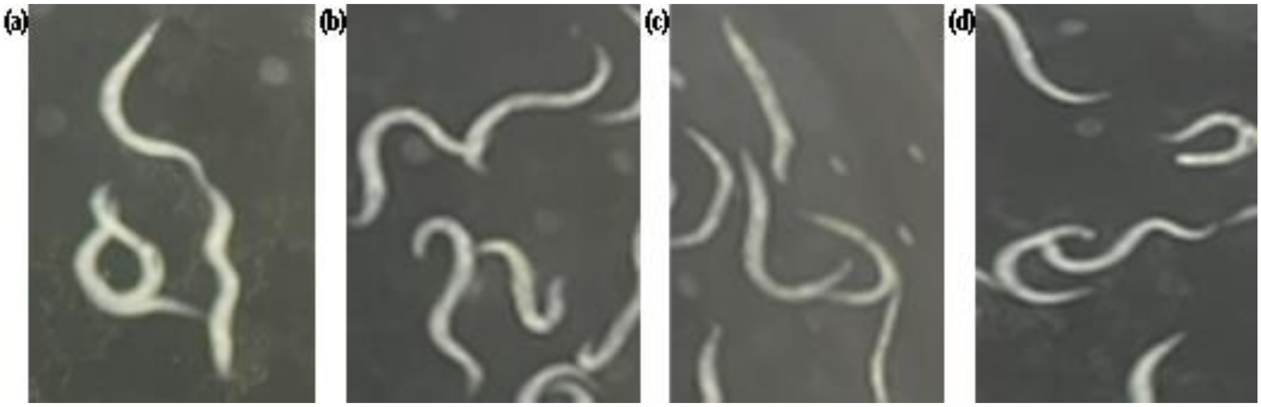

Figure S7. In-vivo toxicity of PG and Br-derivatives using *C. elegans* model system. Nematodes were treated with 50 μg/mL of: (**a**) PG; (**b**) PG-Br; (**c**) $PG-Br_2$; (**d**) DMSO. Images were taken under Stereomicroscope Carl Zeiss™ Stemi 508 (Zeiss Group, Germany) at 40× magnification.

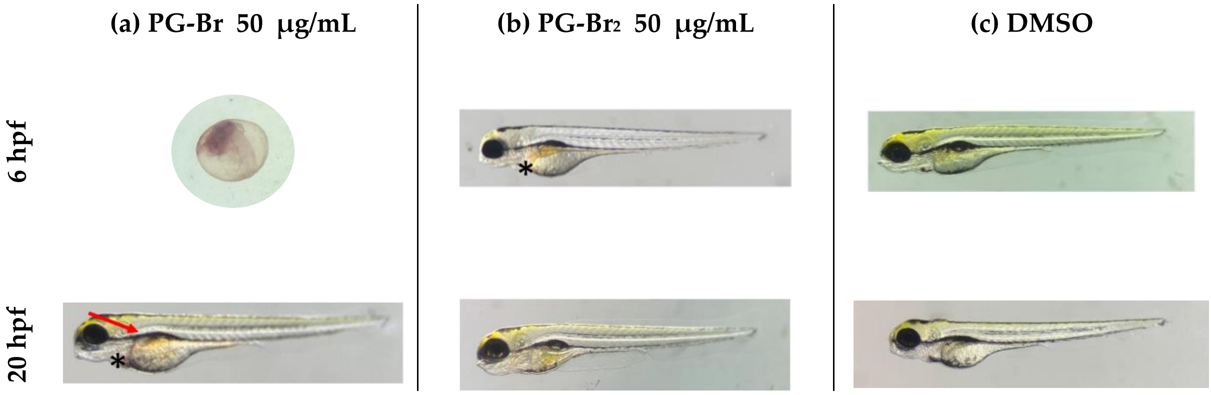

Figure S8. Effects on zebrafish from the treatment of higher concentrations of 50 μg/mL of: (**a**) PG-Br; (**b**) $PG-Br_2$, compared to (**c**) DMSO control. Images were taken under a stereomicroscope (SMZ-143-N2GG, Motic, Germany) at 3.5× magnification. Red arrow (→) points to abnormal liver and asterisk (*) denotes abnormal heart.
